# Worldwide socioeconomic status and stroke mortality: an ecological study

**DOI:** 10.1186/1475-9276-12-42

**Published:** 2013-06-15

**Authors:** Sheng Hui Wu, Jean Woo, Xin-Hua Zhang

**Affiliations:** 1 Division of Epidemiology, Department of Medicine, Vanderbilt University Medical Center, Nashville, TN, USA; 2 Department of Medicine and Therapeutics, The Chinese University of Hong Kong, Hong Kong, SAR, China; 3 Beijing Hypertension League Institute, Beijing, China

**Keywords:** Socioeconomic status, Stroke, Mortality

## Abstract

**Introduction:**

The effect of socioeconomic status (SES) on stroke mortality at population level has been controversial. This study explores the association of SES in childhood and adulthood with stroke mortality, as well as variations in this association among countries/regions.

**Methods:**

Sex-specific stroke mortality at country level with death registry covering ≥ 70% population was obtained from the World Health Organization. Human Development Index (HDI) developed by the United Nations was chosen as the SES indicator. The associations between the latest available stroke mortality with HDI in 1999 (adulthood SES) and with HDI in 1960 (childhood SES) for the group aged 45–54 years among countries were examined with regression analysis. Age-standardized stroke mortality and HDI during 1974–2001 were used to estimate the association by time point.

**Results:**

The population data were available mostly for low-middle to high income countries. HDI in 1960 and 1999 were both inversely associated with stroke mortality in the group aged 45–54 years in 39 countries/regions. HDI in 1960 accounted for 37% of variance of stroke mortality among countries/regions; HDI in 1999 for 35% in men and 53% in women (*P* < 0.001). There was a quadratic relationship between age-standardized stroke mortality and HDI for the countries from 1974 to 2001: the association was positive when HDI < 0.77 but it became negative when HDI > 0.80.

**Conclusions:**

SES is a strong predictor of stroke mortality at country level. Stroke mortality increased with improvement of SES in less developed countries/region, while it decreased with advancing SES in more developed areas.

## Introduction

Stroke causes 5.5 million deaths and the loss of 49 million disability-adjusted life years worldwide each year [1]. It has been estimated that by the year 2020 stroke will remain the second leading cause of death, and in terms of disability it will be among the five most important causes of disability worldwide [2].

It has been suggested that stroke burden and time trend of stroke varied among populations [3,4], but trends in common risk factors may not fully explain time or inter-population variations in stroke occurrence [5-10]. According to Global Health Statistics in 1996, the higher stroke mortality rates for men aged 45–59 years were observed in Formerly Socialist Economies of Europe (FSE, 135.5/100,000), China (CHN, 134.3), Latin America and the Caribbean (LAC, 104.1). The lower rates were reported from countries with Established Market Economies (EME, 36.9, such as USA, Canada, Japan and Australia); Middle Eastern Crescent (MEC, 73.2) and Other Asia and Islands (OAI, 85.7). In women, the higher rates were observed in CHN (110.5), LAC (83.9) and FSE (83.1). The lower rates were in EME (22.7), MEC (65.5) and OAI (67.2) [3]. Sarti and Rastenyte presented trends of stroke mortality during 1968–1994 [4]. The most developed countries experienced remarkably declining trends about 20 years, but less developed countries like eastern European countries experienced increasing trends for stroke mortality during the same period. Recent studies on life course exposures demonstrated that children exposed to low socioeconomic status (SES) were at high risk of stroke at adulthood [11-13]. The findings from adult population studies examining the relationship between SES and the risk of stroke are conflicting, with some reporting positive and some negative associations [14-17]. The effect of population SES on stroke mortality at country level has not been addressed. We carried out an ecological study to explore the association between SES in childhood and in adulthood with stroke mortality among countries/regions, to help define populations at a higher risk and predict the trends of stroke mortality for health policy.

## Methodology

### Data sources

The inclusion criteria for countries/regions included in our study were the estimated coverage of all deaths reported in the routine mortality statistics for countries/regions ≥ 70% [18], and availability of data on stroke mortality and SES indicator. The data included deaths for broad stroke categories registered in national vital registration systems, with underlying cause of death as coded by the relevant national authority (ICD 8–9 code: 430–438 and ICD 10 code: I60-69).

Infant mortality rates and children under 5 mortality rates are widely used as measures of social development [19,20], while Gross Domestic Product (GDP)/capita most directly measures material circumstances, the Human Development Index (HDI) is a complex index of socioeconomic status at population level [21-23], they were all compared in the current study.

The HDI is an index combining normalized measures of life expectancy, literacy, educational attainment, and GDP per capita at country level [22]. The index, the development of which had been influenced by the ideas of Indian Nobel Prize winner Amartya Sen, has been used since 1990 by the United Nations in its annual Human Development Report [22]. The HDI combines three basic dimensions:

● Life expectancy at birth, as an index of population health and longevity.

● Knowledge and education, as measured by the adult literacy rate (with two-thirds weighting) and the combined primary, secondary, and tertiary gross enrollment ratio (with one-third weighting).

● Standard of living, as measured by the natural logarithm of gross domestic product (GDP) per capita at purchasing power parity (PPP) in United States dollars.

Performance in each dimension is expressed as a value between 0 and 1, the higher the number, the better the result. Therefore, HDI might be expected to indicate socioeconomic development more comprehensively.

The above-mentioned indicators were used to explore the associations between SES and stroke mortality at population level based on the availability of the data, and the optimal one was selected to indicate SES in this study.

The earliest available and accessible SES indicator was in 1960. Populations in childhood in 1960 and dying of stroke in their adulthood most likely happened over their 35 years old. But mortality rate from stroke in the age group of 35-44 years was very low therefore fluctuated significantly from year to year. Mortality rate for the age group of 45–54 years old was used to analyze the association between stroke mortality and SES in childhood and adulthood. Sex-specific stroke mortality rates (per 100,000 per year) for the age group of 45–54 years in the latest available 3 years (1997–2003) were averaged using data derived from the World Health Organization (WHO) [6]. In the present study, SES in childhood was represented by HDI in 1960, infant mortality rate in 1960, under-5 mortality rate in 1960 and GDP/capita in 1970, which were all the earliest available and accessible data. Since the latest available data for stroke mortality ranged between 1997 and 2003, and SES indicators in adulthood were obtained in 1999 or 2000 for most of the countries/regions, SES in adulthood was represented by HDI in 1999, GDP/capita in 1999, infant mortality rate in 2000 and under-5 mortality rate in 2000.

In order to minimize confounding effects, we used the data on systolic blood pressure, prevalence of smoking and obese, alcohol and saturated fat consumption as controlling variables in this analysis. Data for adult mean systolic blood pressure (SBP) in 2002, prevalence of obese (OB) in the latest available year (1995–2003), per capita alcohol consumption (> 15 years) (in litres of pure alcohol) in 2000–2001 (ALC), were obtained from WHO data sources [6,7,24,25].

Countries/regions with data available on stroke mortality (1974–1976, 1979–1981, 1984–1986, 1989–1991, 1994–1996 and 1999–2001) and HDI (1975, 1980, 1985, 1990, 1995 and 2000) calculated through the same method were used to analyze the effect of socioeconomic development on stroke mortality. Sex-specific stroke mortality rates (per 100,000 per year) in eight age classes, 35–39 years, 40–44 years, 45–49 years, 50–54 years, 55–59 years, 60–64 years, 65–69 years and 70–74 years from 1975 to 2000 for the countries/regions meeting the inclusion criteria were derived from WHO [6]. The sex-specific stroke mortality rate in 5 year age group (35–74 years) were smoothed over 5 years at each of six time periods, and then standardized to 35–74 years according to the world standard population (1996), respectively [26]. The age range 35–74 years was used, because death from stroke rarely occurs in people younger than 35, and diagnostic accuracy on death certificates for stroke may be less reliable for people older than 74 years [27].

### Statistical analysis

Since the mortality data did not follow a normal distribution, they were transformed by natural logarithm prior to analysis.

Pearson correlation analysis was made between stroke mortality and childhood SES indicators (HDI in 1960, infant mortality in 1960, under-5 mortality in 1960 and GDP/capita in 1970), and between stroke mortality and adulthood SES indicators (HDI in 1999, GDP/capita in 1999, infant mortality in 2000 and under-5 mortality in 2000), as well as between childhood or adulthood SES indicators.

Linear regression models were constructed with stroke mortality as dependent variable, and SES indicators in childhood and adulthood as respective independent variables. The choice of SES indicators was identified by comparing the sum of ranks of correlation coefficients between each childhood or adulthood indicator and stroke mortality, and the higher the correlation coefficient, the lower the rank.

Linear regression models were constructed with stroke mortality as the dependent variable, and HDI in 1960 and HDI in 1999 as independent variables. Multiple regression models were constructed with stroke mortality as the dependent variable, and HDI in 1960 and HDI in 1999 as independent variables plus confounding variables. Since the sample size was not large enough, confounding variables were adjusted one by one separately.

Curve estimation was used to explore the association between HDI and stroke mortality when each country/region at each of six time periods was regarded as one study unit and considered simultaneously. X axis represents HDI for each country/region at each of six time periods, and Y axis represents the corresponding stroke mortality. The quadratic model identified had better goodness-of-fit (larger R square) than linear, logarithmic, inverse, cubic, compound, power, S, growth, exponential and logistic models in this study, therefore, only the results of quadratic model were showed.

The significance level was put at *P* < 0.05 (2-tailed). SPSS software (version 16.0, copyright SPSS Inc. in USA) was used for the statistical analysis.

The HDI in 1980, 1985, 1990 and 1995 were also used to explore the association of stroke mortality in the latest available year with the SES indicator.

## Results

Countries/regions with the HDI and some confounding factors used in the analysis lists in Table 1. The status of socioeconomic development among the 39 countries/regions ranged from HDI as low as 0.70 in Slovakia to HDI as high as 0.939 in Norway in 1999. Many countries/regions had no nationwide survey data of prevalence of hypertension or obesity. Stroke mortality rate was high in Mauritius (98.5 in men and 56.1in women) and low in Switzerland (7.7 per 100,000 in men and 7.5 in women) in recent years.

**Table 1:** **The Human Development Index** (**HDI**) **in 1999 and the distribution of some available confounding factors for the countries**/**regions involved in the study**

Country/region*	**Stroke mortality (1/100,000), 45–54 yrs**		**HDI 1999**	**SBP**		**HPT (%)**		**SMK (%)**		SF**	ALC**	**OB (%)**	
	**Men**	**Women**		**Men**	**Women**	**Men**	**Women**	**Men**	**Women**			**Men**	**Women**
**South America**													
ARG	59.83	34.30	0.842	120	119	..	..	46.8	34.0	4.45	8.55	..	..
BRA	66.00	49.37	0.750	124	119	..	..	38.2	29.3	3.10	5.30	8.9	13.1
CHL	30.37	25.13	0.825	119	116	..	..	26.0	18.3	2.41	6.02	19.0	25.0
COL	30.23	31.40	0.765	122	119	..	..	22.3	23.5	6.62	5.90	..	..
CRI	15.20	17.60	0.821	122	117	..	..	28.6	6.6	8.85	5.50	..	..
ECU	28.87	23.73	0.726	124	122	..	..	45.5	17.4	17.63	2.00	..	..
SLV	20.13	15.30	0.701	..		..	..	38.0	12.0	3.98	3.50	..	..
MEX	23.47	20.20	0.790	125	121	38.6	30.1	51.2	18.4	4.60	4.62	18.6	28.1
PAN	24.37	17.63	0.784	..		..		56.0	20.0	6.55	6.00	..	..
PRY	40.10	50.37	0.738	122	128	32.4	41.9	24.1	5.5	5.81	6.70	..	..
VEN	40.93	34.30	0.765	120	117	47.7	32.2	41.8	39.2	4.35	8.80	..	..
URY	45.63	37.67	0.828	..		..		31.7	14.3	3.04	7.00	17.0	19.0
**Mean**	**35.43**	**29.75**	**0.778**	**122**	**120**	**39.6**	**34.7**	**37.5**	**19.9**	**5.95**	**5.82**	**15.9**	**21.3**
**Asia**													
HKG	20.37	11.50	0.880	130	123	..	..	27.1	2.9	..	..	..	..
ISR	10.23	7.13	0.893	128	121	..	..	33.0	24.0	8.87	2.00	19.8	25.4
KOR	53.83	26.23	0.875	126	121	21.8	19.4	65.1	4.8	5.88	7.70	1.7	3.0
JPN	36.87	18.07	0.928	127	119	42.7	35.0	52.8	13.4	2.83	7.38	..	…
SGP	20.57	12.03	0.876	124	119	..	..	26.9	3.1	..	2.73	5.3	6.7
**Mean**	**28.37**	**14.99**	**0.890**	**127**	**121**	**32.3**	**27.2**	**41.0**	**9.6**	**5.86**	**4.95**	**8.9**	**11.7**
**Africa**													
MUS	98.53	56.07	0.765	127	124	..	..	44.8	2.9	3.51	3.20	8.0	20.0
**Europe**													
AUT	17.10	13.10	0.921	129	122	..	..	30.0	19.0	19.49	12.58	..	..
BEL	18.13	13.40	0.935	127	119	..	..	30.0	26.0	34.16	10.06	10.3	11.0
DEN	21.90	11.90	0.921	122	115	..	..	32.0	29.0	22.59	11.93	..	..
FIN	25.63	16.63	0.925	131	125	..	..	27.0	20.0	12.86	10.43	20.8	23.9
FRA	15.93	8.13	0.924	129	125	..	..	38.6	30.3	19.79	13.54	..	..
DEU	15.43	10.67	0.921	134	130	55.4	56.6	39.0	31.0	24.68	12.90	13.6	12.3
GRC	26.10	14.43	0.881	131	124	18.5	15.9	47.0	29.0	3.57	9.30	..	..
HUN	66.67	29.10	0.829	134	126	86.2	..	44.0	27.0	24.03	11.92	18.4	20.4
IRL	16.70	14.70	0.913	..		..	..	32.0	31.0	17.58	14.50	14.0	12.0
ITA	15.27	9.97	0.909	129	122	42.0	43.3	32.4	17.3	12.96	9.14	..	…
NLD	14.47	15.83	0.931	131	122	..	..	37.0	29.0	14.68	9.74	10.2	11.9
NOR	13.67	9.60	0.939	..		..	..	31.0	32.0	17.34	5.80	6.8	5.8
PRT	38.73	20.57	0.874	127	124	..	..	30.2	7.1	15.70	12.49	..	..
ESP	17.20	9.23	0.908	123	118	41.7	39.0	42.1	24.7	5.42	12.25	12.3	12.1
SWE	15.70	11.37	0.936	131	125	39.6	40.9	19.0	19.0	18.39	6.90	10.4	9.5
CHE	7.70	7.53	0.924	126	115	..	..	39.0	28.0	12.57	11.53	7.9	7.5
GBR	17.93	15.20	0.923	132	127	34.7	25.7	27.0	26.0	8.70	10.39	..	..
**Mean**	**21.43**	**13.61**	**0.913**	**129**	**123**	**45.4**	**36.9**	**34.0**	**25.0**	**16.74**	**10.91**	**12.5**	**12.6**
**USA,CAN,AUS &NZL**													
USA	17.70	14.37	0.934	123	119	21.0	19.7	25.7	21.5	6.37	8.51	25.8	
CAN	9.93	8.93	0.936	126	118	23.5	15.6	27.0	23.0	16.37	8.26	15.9	31.8
AUS	9.57	7.77	0.936	118	125	30.8	20.1	21.1	18.0	12.75	9.19	14.8	13.9
NZL	14.27	14.03	0.913	134	123	..	..	25.0	25.0	14.17	9.80	21.9	15.3
**Mean**	**12.87**	**11.28**	**0.930**	**125**	**121**	**25.1**	**18.5**	**25.0**	**22.0**	**12.42**	**8.94**	**19.6**	**23.2**

*ARG Argentina, *AUS* Australia, *AUT* Austria, *BEL* Belgium, *BRA* Brazil, *CAN* Canada, CHE, Switzerland, CHL, Chile; COL, Colombia; CRI, Costa Rica, DEU, Germany; *DNK* Denmark; *ECU* Ecuador, *ESP* Spain, *FIN* Finland, *FRA* France, *GRC* Greece, *GBR* United Kingdom, *HKG* Hong Kong, *HRV* Costa Rica, *HUN* Hungary, *IRL* Ireland, *ITA* Italy, I*SR* Israel, *JPN* Japan, *KOR* Korea, Rep. of; *MUS* Mauritius, *MEX* Mexico, *NLD* Netherlands, *NOR* Norway, *NZL* New Zealand, *PAN* Panama *PRT* Portugal, *PRY* Paraguay, *SGP* Singapore *SLV* El Salvador, *SWE* Sweden, *USA* United States of America, *VEN* Venezuela, *URY* Uruguay.

*OB* Adults (≥15 years) who are obese (%) in the latest available year.

*SBP* Mean adult SBP (mmHg) in 2002.

*HPT* The latest available hypertension prevalence.

*SMK* The latest available adult smoking prevalence.

*SF* Saturated fat (/capita/year (kg)) in 1999.

*ALC* Per capita alcohol consumption (>15 years) (in litres of pure alcohol) in 2000–2001.

** Data available irrespective of men and women.

Pearson correlation analysis among SES indicators in childhood and adulthood shows that the indicators were correlated each other (Table 2). The choice of SES indicators was determined by comparing sum of ranks of correlation coefficients between each indicator and stroke mortality (Table 3). The correlation coefficients and the rank of the correlations were very close for GDP/capita (slightly higher in childhood) and HDI (slightly higher in adulthood) for both 1960 and 1999. HDI was chosen because it was a more comprehensive indicator of socioeconomic development status. HDI consisted of not only economic productivity but also life-expectancy, health care services and education levels. Those factors are largely related to the susceptibility to development of stroke and efficiency in primary and secondary prevention of stroke in the populations.

**Table 2:** Pearson correlation coefficients between socioeconomic status indicators in childhood and adulthood

**SES indicators**	**GDP/capita in 1970**	**Infant mortality in 1960^**	**Under**-**5 mortality in 1960^^**
**HDI in 1960**	*r* = 0.860	*r* = −0.871	*r* = −0.875
	*P* = 0.000	*P* = 0.000	*P* = 0.000
**GDP/capita in 1970**		*r* = −0.684	*r* = −0.680
		*P* = 0.000	*P* = 0.000
**Infant mortality in 1960^**			*r* = 0.990
			*P* = 0.000
	**HDI in 1999**	Infant mortality in 2000*	**Under**-5 mortality in 2000**
**GDP/capita in 1999**	*r* = 0.876	*r* = −0.139	*r* = −0.731
	*P* = 0.000	*P* = 0.411	*P* = 0.000
**HDI in 1999**		*r* = −0.131	*r* = −0.922
		*P* = 0.440	*P* = 0.000
Infant mortality in 2000*			*r* = 0.728
			*P* = 0.000

*CC* correlation coefficient.

P significance.

^ Infant mortality in 1960 (per 1,000 live births).

^^ Under-5 mortality in 1960 (per 1,000).

* Infant mortality rate (per 1,000 live births).

** Under-5 mortality rate (per 1,000).

**Table 3:** **The sum of rank of correlation coefficients between childhood ses indicators and stroke mortality in men and women aged 45**–**54 years**

**SES indicators**	**Stroke mortality^**				
	**Men**		**Women**		**Sum of rank**
	**CC**	**Rank**	**CC**	**Rank**	
**HDI in 1960**	−0.612*	**2**	−0.608*	**2**	**4**
**GDP/capita in 1970**	−0.623*	**1**	−0.620*	**1**	**2**
Infant mortality in 1960^^	0.517*	3	0.585*	3	6
Under-5 mortality in 1960^^^	0.490*	4	0.575*	4	8
**GDP/capita in 1999**	−0.594*	**1**	−0.700*	**2**	**3**
**HDI in 1999**	−0.594*	**1**	−0.727*	**1**	**2**
Infant mortality in 2000^^	0.174	4	0.053	4	8
Under-5 mortality in 2000^^^	0.381*	3	0.587*	3	6

*SES* socioeconomic status.

*CC* correlation coefficient.

^ Per 100,000 per year (Ln).

* *P* < 0.05.

^^ Infant mortality (per 100,000).

^^^ Under-5 mortality (per 1,000) in 1960.

**Figure 1:**
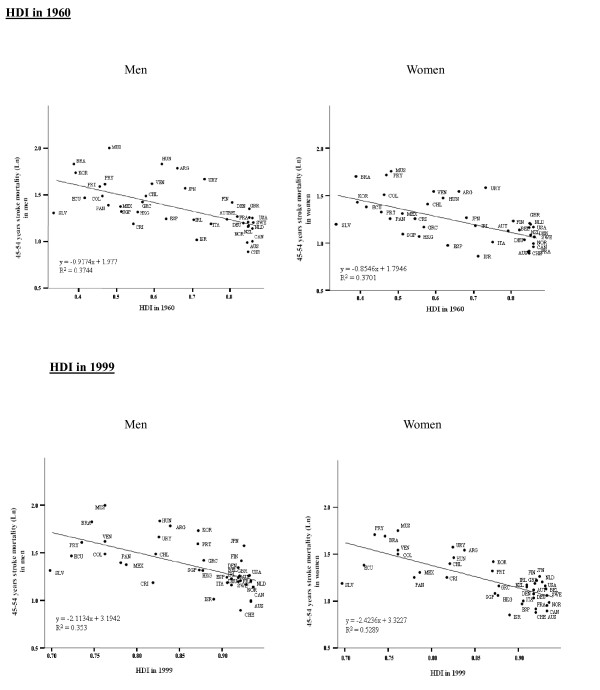
**Linear regression between stroke mortality (per 100,000 per year)in the latest available three years and HDI in 1960 and HDI in 1999 in the group aged 45**–**54 years.** Abbreviations: *ARG* Argentina, *AUS* Australia, *AUT* Austria *BEL* Belgium *BRA* Brazil, *CAN* Canada, *CHE* Switzerland, *CHL* Chile, *COL* Colombia, *CRI* Costa Rica, *DEU* Germany, *DNK* Denmark, *ECU* Ecuador, *ESP* Spain, *FIN* Finland, *FRA* France, *GRC* Greece, *GBR* United Kingdom, *HKG* Hong Kong, *HRV* Costa Rica, *HUN* Hungary, *IRL* Ireland, *ITA* Italy, *ISR* Israel, *JPN* Japan, *KOR* Korea, Rep. of; *MUS* Mauritius, *MEX* Mexico, *NLD* Netherlands, *NOR* Norway, *NZL* New Zealand; *PAN* Panama, *PRT* Portugal, *PRY* Paraguay, *SGP* Singapore, *SLV* El Salvador, *SWE* Sweden, *USA* United States of America, *VEN* Venezuela, *URY* Uruguay.

Table 4 and Figure 1 show that both HDI in 1960 and HDI in 1999 were inversely associated with stroke mortality in men and women. HDI in 1960 explained 37% of variance of stroke mortality among countries/regions in men and women (*P* < 0.001). HDI in 1999 explained 35% in men and 53% in women (*P* < 0.001), respectively. Table 4 shows that HDI in 1960 and HDI in 1999 was still significantly and negatively correlated with stroke mortality in men and women, respectively after adjusting for each confounding variable.

**Table 4:** **Regression analysis of log**-**stroke mortality in the latest available three years in the group aged 45**–**54 years with HDI in 1960 and HDI in 1999** (**Unadjusted and Adjusted Models**)

**HDI and confounding variables**	Stroke mortality* (men)			Stroke mortality* (women)		
	**RC**	***P***	**Adjusted*R***^***2***^	**RC**	***P***	**Adjusted*R***^***2***^
**Unadjusted model HDI in 1960**	−**0**.**917**	**0**.**000**	**0**.**357**	−**0**.**855**	**0**.**000**	**0**.**353**
Adjusted models** **HDI in 1960**+						
SBP	−1.147	0.000	0.446	−1.023	0.000	0.444
HPT	−1.163	0.000	0.629	−1.039	0.005	0.443
SMK	−0.784	0.001	0.380	−0.945	0.000	0.349
Obese	−1.440	0.000	0.518	−1.057	0.001	0.439
ALC	−1.071	0.000	0.370	−0.906	0.000	0.360
SF	−0.910	0.001	0.367	−0.807	0.001	0.411
**Unadjusted model HDI in 1999**	−**2.113**	**0.001**	**0.336**	−**2.424**	**0.000**	**0.516**
Adjusted models** **HDI in 1999**+						
SBP	−2.794	0.000	0.412	−2.945	0.000	0.640
HPT	−2.190	0.017	0.314	−2.964	0.000	0.670
SMK	−1.831	0.000	0.400	−2.411	0.000	0.503
Obese	−4.018	0.000	0.625	−3.623	0.000	0.663
ALC	−2.561	0.000	0.348	−2.765	0.000	0.527
SF	−1.912	0.002	0.325	−2.203	0.000	0.519

*RC* regression coefficient.

*SBP* mean adult SBP in 2002.

*OB* adults (≥ 15 years) who are obese (%) in the latest available year.

*HPT* the latest available hypertension prevalence.

*SMK* the latest available adult smoking prevalence.

*SF* saturated fat (/capita/year (kg)) intake in 1999.

*ALC* per capita alcohol consumption (> 15 years) (in litres of pure alcohol) in 2000–2001.

* Per 100,000 per year (Ln).

** Adjusted model: each adjustment only adjusted for one confounding variable.

Stroke mortality in the countries/regions at the bottom tertile of HDI both in 1960 and 1999 was 2.52 and 2.61 times higher than those at the top tertile of HDI in both periods in men (38.5 per 100,000 per year, 95% CI: 15.5-61.6 vs. 14.8, 10.9-18.7), and women (28.8, 14.2-43.4 vs. 11.4, 8.5-14.4) separately.

**Figure 2:**
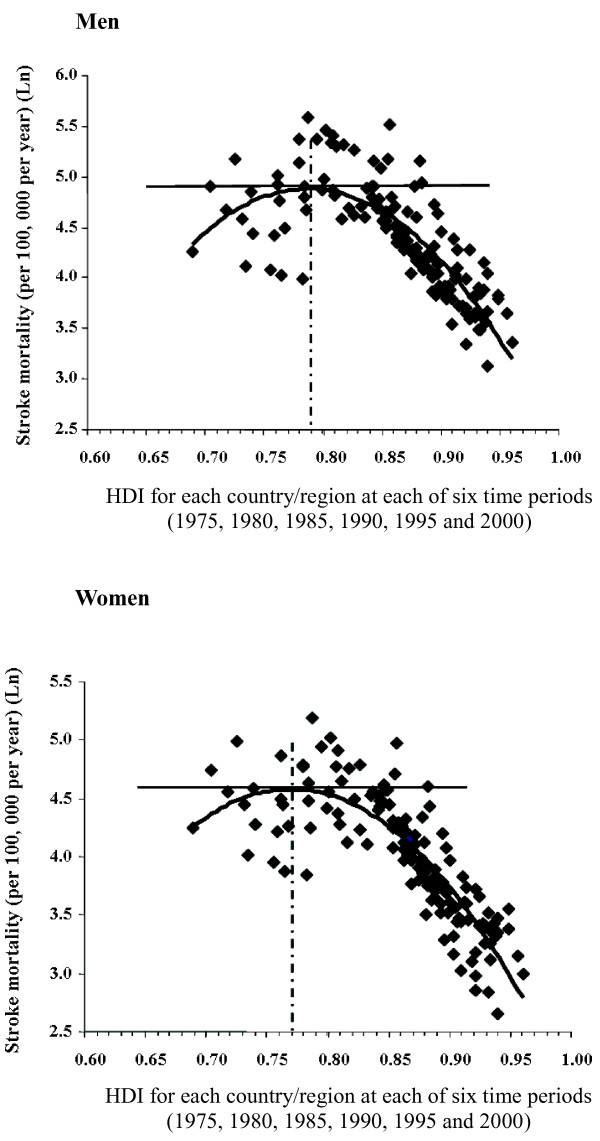
**Curve fit for age-standardized stroke mortality (35**-**74 years)and HDI when each.** Country/region at each time period as one study unit.

Figure 2 illustrates that the peak of age-standardized stroke mortality was exhibited at about HDI = 0.77-0.81 for men and 0.75-0.79 for women (*P* < 0.01). Stroke mortality increased with improvement of HDI if HDI < 0.81 for men and HDI < 0.79 for women, while stroke mortality decreased with increasing HDI if HDI > 0.77 for men and HDI > 0.75 for women during 1974–2001 when each country/region at each of six time periods was considered simultaneously. The strength of the association was stronger as the year of the SES indicatorbecame closer to the year of mortality data used (see Additional file 1: Table S1).

## Discussion

This ecological study quantitatively estimated the effect of SES on stroke mortality at population level. HDI was used as a SES indicator in this study due to its significant association with stroke mortality and more comprehensively represented the social and economic development status at country level. In general, both childhood SES (HDI in 1960) and adulthood SES (HDI in 1999) were inversely associated with stroke mortality in recent years for men and women in middle- or high-income countries/regions. The direction of the association between SES and stroke mortality was opposite for low and high HDI levels: the association was positive for the countries/regions at low HDI level, but it was negative for the countries/regions at high HDI level (p < 0.01 for both directions).

Our finding that the risk of stroke is associated with low SES in childhood at population level suggests that exposure to socioeconomic deprivation in early life might increase the risk of stroke in adulthood. Possible pathophysiological mechanisms may include underlying lasting changes in vascular structure in response to under-nutrition in early life, activation of the renin-angiotensin system predisposing to the development of hypertension [28,29], impact on hormonal and metabolic programming resulting in insulin resistance and diabetes [30,31], adverse lipid and thrombogenic profiles [32,33] predisposing to atherogenesis [11,34].

A quadratic relationship revealed that the risk of stroke increased with improvement of SES in regions at a lower stage of socioeconomic development, while it decreased with increasing SES in regions at a higher stage of socioeconomic development. This finding likely reflected the changing pattern lifestyle, health literacy and access to health services through different stages of socioeconomic development. High blood pressure, obesity and hyperlipidemia are risk factors for stroke that are related to an unhealthy lifestyle pattern consisting of energy-dense diet, physical inactivity, smoking, and alcohol use. Such high risk profiles have been documented to be negatively associated with SES in middle- or high-income countries/regions [35-40], but positively associated with SES in low-income countries [41-44]. SES may affect health status through lifestyle choices [45], and healthy lifestyle was associated with lower risk of stroke [46,47]. SES also related to education and health literacy in populations, which in turn related to a lower risk of stroke [48,49]. Finally, SES is related to the accessibility and quality of health care services which are directly related to the prevention and treatment of stroke [24,49]. The association between stroke mortality and the HDI in the same time period was stronger than the association of stroke mortality with the HDI in an earlier time period for the same cohort. This may suggest that health care services play a more important role for the prognosis of stroke patients.

Our study examined the association between the risk of stroke and SES among countries/regions. In agreement with our results, some studies reported inverse associations between SES and stroke mortality in middle- or high-income countries/regions [14,15]. Studies from USA and northern European countries in the 1980s reported high stroke mortality in people with manual occupations and low mortality in the non-manual class [50]. An analysis from the 1990s assessed stroke mortality by educational level in ten European countries based on longitudinal data [51]. For all countries in all age groups, higher mortality rates were reported in groups with an education below upper secondary or equivalent. This was consistent for men (relative risk 1.27, 95% CI 1.24-1.30) and women (1.29, 1.27-1.32). In contrast, observations from low-income countries showed that populations with high socioeconomic status were at a higher risk of stroke compared with those with low socioeconomic status [16,17]. For instance, stroke mortality was higher in urban compared with rural Tanzania, there being a graded response depending on wealth. The yearly age-adjusted rates per 100,000 in the group aged 15–64 year for the three project areas (urban, fairly prosperous rural, and poor rural, respectively) were 65 (95% CI: 39–90), 44 (31–56), and 35 (22–48) for men, and 88 (48–128), 33 (22–43), and 27 (16–38) for women, respectively [17]. Our study indicated the high correlation between childhood and adulthood SES and therefore we could not determine if childhood/adulthood SES related with stroke risk independently of adulthood/childhood SES due to their colinearity. However, we found that stroke mortality in the countries/regions at the bottom tertile of HDI both in 1960 and 1999 was 2.52 and 2.61 times higher than those at the top tertile of HDI in both periods.

There are limitations in our study. Given the nature of the ecological study design, our results could be biased by the ecological fallacy. We could not completely rule out the possibility of residual confounding due to unmeasured or inadequately measured covariates. The results from the population level could not be directly applied to individual patients. HDI was a comprehensive country level index estimated with comparable information; therefore, the bias in SES measurement was limited. Data on stroke mortality is not available for very low-income countries/regions, so that the results only applied to low to middle or high income countries/regions. Stroke mortality at different time points could be affected by the changing of ICD codes system over time. In the present study, the data available comprise deaths for stroke registered in national vital registration systems, with underlying cause of death as coded by the relevant national authority. Moreover, our analyses are restricted to overall category of stroke which is essentially similar in the 8th and 9th, and 9th and 10th [4,52,53], and for which death certificate data are more accurate than for specific diseases. The accuracy and consistency over time and between regions in the diagnosis may affect the comparison of stroke mortality at different time periods among countries/regions. However, the concerns about diagnostic accuracy in these statistics are minimized by inclusion of all cerebrovascular disease for analysis, thus improving comparability across countries and time. We did not analyze subtypes of stroke mortality due to the unavailability of the data, and the study is not able to examine regional differences and time trends in mortality from different stroke subtypes. Different SES indicators can establish groups with differential exposures and identify specific as well as generic mechanisms relating SES to health [54]. There is no best single indicator. Heath is captured in HDI, so it could be a limitation to study HDI and health outcomes. However, HDI is a composite measure of the three factors key in allowing people to lead more fulfilling lives and strong association with stroke mortality. Therefore, it was used as the SES indicator in this study. It should be mentioned that there was no log-linear or quadratic linear association between HDI and mortality from other cardiovascular/vascular diseases were observed (data not shown here).

In conclusion, SES is an important population indicator of stroke mortality risk. From low to low-middle income countries/regions, stroke mortality increases with improvement of SES (measured by HDI). From middle to high income countries/regions, stroke mortality decreases with advancing SES. In low SES environment, advantaged populations are at high risk of stroke. In high SES environment, disadvantaged populations are at high risk of stroke. For the countries/regions experiencing transition, the high and low risk populations are under epidemiological transition. This information allows health policy makers to mobilize appropriate resources and identify target populations for more efficient health protection and better health care services.

## Abbreviations

ARG: Argentina; AUS: Australia; AUT: Austria; BEL: Belgium; BGR: Bulgaria; BP: blood pressure; BRA: Brazil; CAN: Canada; CC: correlation coefficient; CHE: Switzerland; CHL: Chile; CHN: China; COL: Colombia; CRI: Costa rica; CZE: Czech republic; DEN: Denmark; DEU: Germany; ECU: Ecuador; ESP: Spain; FIN: Finland; FRA: France; FSE: Formerly socialist economies of Europe; GBR: United Kingdom; GDP: Gross domestic product; GRC: Greece; HDI: Human development index; HDR: Human development report; HKG: Hong kong; HPT: Prevalence of hypertension; HUN: Hungary; ICD: International classification of diseases; IFD: Infectious disease; IFD (‰): Permillage of infectious disease death/total deaths; IHD: Ischemic heart disease; IRL: Ireland; ISR: Israel; ITA: Italy; JPN: Japan; KOR: Korea republic of; LAC: Latin America and the caribbean; MEC: Middle eastern crescent; MEX: Mexico; M/F: Male/Female; MONICA: Multinational monitoring of determinants and trends in cardiovascular disease; MUS: Mauritius; NLD: Netherlands; NOR: Norway; NZL: New Zealand; OAI: Other Asia and islands; OB: Obese; PAN: Panama; POL: Poland; PRT: Portugal; PRY: Paraguay; RC: Regression coefficient; ROU: Romania; SBP: Systolic blood pressure; SD: Standard deviation; SES: Socioeconomic status; SF: Saturated fat; SGP: Singapore; SLV: El salvador; SMK: Smoking; SMRs: Standardized mortality ratios; SWE: Sweden; TC: Total cholesterol; TG: Triglycerides; UN: The United Nations; UNDP: The United Nations Development Program; URY: Uruguay; USA: United states of america; VEN: Venezuela; VS: Versus; WHO: The World Health Organization.

## Competing interests

The authors declare that they have no competing interests.

## Author contributions

XZ designed the study; SHW, XZ and JW conducted the research; SHW analyzed the data and drafted the paper; XZ had primary responsibility for the final content. All authors read and approved the final manuscript.

## Supplementary Material

Additional file 1: Table S1 The association of stroke mortality rate (1/100,000, log scale) in the latest available year with the Human Development Index (HDI) in 1980, 1985, 1990 and 1995 for the age group of 45-54y in men and women. Click here for file
