# Supplementary material for: Worldwide socioeconomic status and stroke mortality: an ecological study
Source: Int J Equity Health. 2013 Jun 15;12:42. doi: 10.1186/1475-9276-12-42 (PMC3695775; doi:10.1186/1475-9276-12-42)
Supplement: Additional file 1: Table S1 — The association of stroke mortality rate (1/100,000, log scale) in the latest available year with the Human Development Index (HDI) in 1980, 1985, 1990 and 1995 for the age group of 45-54y in men and women. [file 1475-9276-12-42-S1.docx]

| **The association of stroke mortality rate (1/100,000, log scale) in the latest available year with the Human Development Index (HDI) in 1980, 1985, 1990 and 1995 for the age group of 45-54y in men and women** | | | | | | | |
| --- | --- | --- | --- | --- | --- | --- | --- |
| **HDIs** | **Stroke mortality (men)** | | |  | **Stroke mortality (women)** | | |
|  | **RC** | ***P*** | **Adjusted *R^2^*** |  | **RC** | ***P*** | **Adjusted *R^2^*** |
| **HDI in 1980** | -1.909 | 0.000 | 0.343 |  | -2.092 | 0.000 | 0.469 |
| **HDI in 1985** | -1.949 | 0.000 | 0.345 |  | -2.191 | 0.000 | 0.481 |
| **HDI in 1990** | -2.047 | 0.000 | 0.348 |  | -2.330 | 0.000 | 0.514 |
| **HDI in 1995** | -2.136 | 0.000 | 0.369 |  | -2.416 | 0.000 | 0.538 |
